# An efficient protocol to enhance recombinant protein expression using ethanol in *Escherichia coli*

**DOI:** 10.1016/j.mex.2015.09.005

**Published:** 2015-10-08

**Authors:** Gaurav Chhetri, Parismita Kalita, Timir Tripathi

**Affiliations:** Molecular and Structural Biophysics Laboratory, Department of Biochemistry, North-Eastern Hill University, Shillong 793 022, India

**Keywords:** High-level protein expression using ethanol, Recombinant protein, Ethanol, *E. coli*, Protein expression, Protein induction, Osmotic stress, Expression of inducible proteins

## Abstract

Bacterial cells can be engineered to express non-native genes, resulting in the production of, recombinant proteins, which have various biotechnological and pharmaceutical applications. In eukaryotes, such as yeast or mammalian cells, which have large genomes, a higher recombinant protein expression can be troublesome. Comparatively, in the *Escherichia coli* (*E. coli*) expression system, although the expression is induced with isopropyl β-d-1-thiogalactopyranoside (IPTG), studies have shown low expression levels of proteins. Irrespective of the purpose of protein production, the production process requires the accomplishment of three individual factors: expression, solubilization and purification. Although several efforts, including changing the host, vector, culture parameters of the recombinant host strain, co-expression of other genes and changing of the gene sequences, have been directed towards enhancing recombinant protein expression, the protein expression is still considered as a significant limiting step. Our protocol explains a simple method to enhance the recombinant protein expression that we have optimized using several unrelated proteins. It works with both T5 and T7 promoters. This protocol can be used to enhance the expressions of most of the proteins. The advantages of this technique are presented below:

• It produces several fold increase in the expression of poorly expressed, less expressed or non-expressed recombinant proteins.
• It does not employ any additional component such as chaperones, heat shock proteins or co-expression of other genes.
• In addition to being inexpensive, easy to manage, universal, and quick to perform, the proposed method does not require any commercial kits and, can be used for various recombinant proteins expressed in the *E. coli* expression system.

## Graphical abstract

**Figure:**
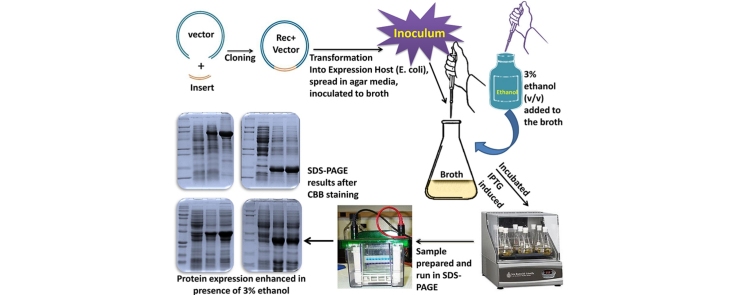

## Method details

### Materials

• High-grade sterile LB agar plates.
• Ethanol (analytical or molecular biology grade).
• Antibiotics (ampicillin, kanamycin, etc.).
• IPTG stock solution (1 M) for protein induction (IPTG should be a filter sterilized).
• Lysis buffer for pellet re-suspension. (Lysis buffer can be phosphate, Tris or PBS buffer of pH 8.0.)
• Properly autoclaved culture vials/tubes for cell growth.
• Shaking incubator for culture growth.
• Cooling centrifuge for cell harvesting.
• Spectrophotometer for monitoring cell growth.
• Erlenmeyer flask of different size depending on the culture volume.
  *Note*: This list does not include any small generic laboratory equipments that are assumed to be available. Chemicals and other components can be used from any reliable company.
• Choice of appropriate antibiotics depends on the vector construct and *E. coli* expression hosts.
  *Note*: Several other vectors and *E. coli* expression hosts are commercially available in the market; they can also be used for molecular cloning and protein expression as per the recommended protocol provided by manufacturers with appropriate selective antibiotics.

## Procedure

### Pilot screening of protein expression in presence of different concentration of ethanol

1. Pick a single colony from the transformed LB agar plate or take 20 μL of glycerol stock and inoculate into 5 mL of LB broth containing appropriate antibiotics according to the vector construct and host cells.
2. Incubate the culture at 37 °C for overnight (14–16 h) at 180 rpm with continuous shaking as starter cultures.
3. Next day take five culture vials and name them as:
  • Control (un-induced).
  • Induced in the absence of ethanol.
  • Induced in the presence of 1% ethanol.
  • Induced in the presence of 2% ethanol.
  • Induced in the presence of 3% ethanol.
4. Inoculate five vials (each containing 5 mL LB medium with appropriate antibiotics) with 20 μL of the overnight grown starter cultures.
5. At the time of inoculation add 1%, 2% and 3% ethanol to the LB medium and grow at 37 °C for a few hours (approx. 3–4 h.) with vigorous shaking, until the OD_600_ reaches 0.5–0.6.
  *Note*: Ethanol should be used in v/v ratio. We have optimized the optimum ethanol concentration and it was observed that 3% ethanol (v/v) gives the maximum enhancement in protein expression. In the presence of more than >5% ethanol cell growth was found to be inhibited.
6. Induce the protein expression by adding IPTG to a final concentration of 1.0 mM.
  *Note*:
  • Do not add IPTG to the culture which will serve as a non-induced control.
  • Minimal IPTG concentration should be optimized in small scale of culture before proceeding to the mass culture.
  • The optimal growth time for TB (Terrific broth) is different from LB (Luria broth) In case of TB OD_600_ should be more than 1.0–1.5 before IPTG induction.
  • In case of auto-induction media the control should be normal LB not the auto-induction media. There is no need to observe the OD, because it does not need the IPTG induction.
7. Grow the cultures for an additional 4–5 h at 37 °C with continuous shaking.
8. Harvest 1 mL of cells from each culture vial by centrifugation for 1 min at 12,000 rpm. Discard the supernatants (remaining media).
9. Re-suspend the cells in 60 μL of buffer (Tris or phosphate or PBS, pH-8.0), 20 μL of 10% SDS & 20 μL of 5X SDS loading dye and lyse by mixing or pipetting.
10. Boil the samples in 100 °C for 5–10 min (Vortex the samples in between).
11. Load the sample in sodium dodecyl sulfate-polyacrylamide gel (SDS-PAGE) and analyze. Screening through pilot experiment is important, which will give the clear picture about the level of over-expression of the protein in the presence of 1%, 2% and 3% ethanol. This will also provide the opportunity to compare the over expression of recombinant protein in the presence and absence of ethanol. SDS-PAGE analysis will support the increase fold of expression with increased percentage of ethanol.

*Note*: Pilot screening can be done at low temperature ranging from 16 °C to 23 °C for 20–22 h after induction with IPTG, depending upon the level of expression and solubility screening of the recombinant proteins. In case of auto-induction media incubate the culture until the OD_600_ reach 0.4–0.6. It is observed that some proteins give higher fold of expression in low temperature. This screening can also provide a clear idea about the level of expression of the protein, which can be helpful for further mass culture. For mass culture setup the experiment according to the same ratio of pilot experiment.

## Method validation

**Fig. 1:**
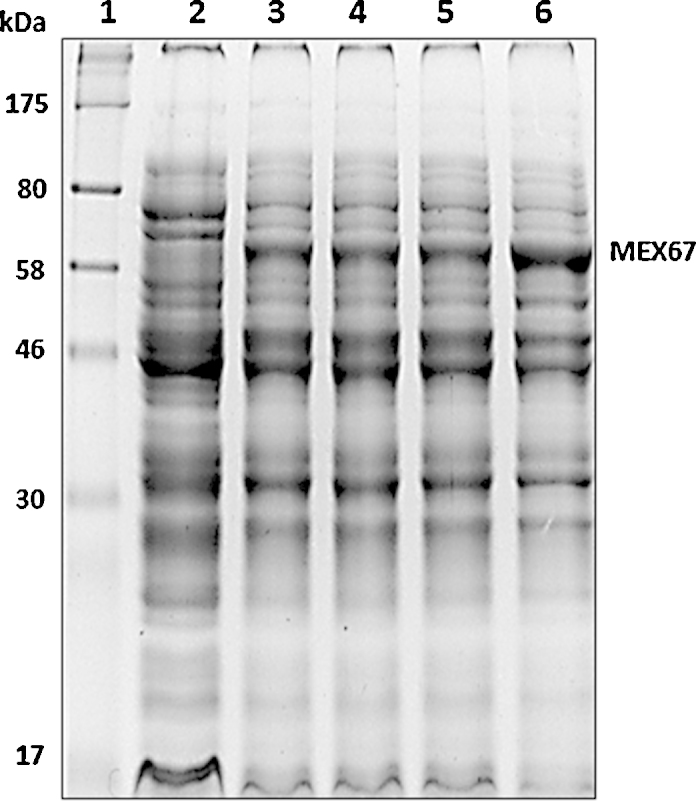
Expression analysis of MEX67 in the presence of osmolytes [5]. Protein samples were separated by 12% SDS-PAGE and stained with CBB. Lane 1, molecular weight markers. Lane 2, MEX67 un-induced control. Lane 3, MEX67 induced in presence of 100 mM glucose. Lane 4, MEX67 induced in presence of 100 mM sorbitol. Lane 5, MEX67 induced in presence of 100 mM mannitol. Lane 6, MEX67 induced in presence of 1% ethanol. The expression was induced with 0.5 mM IPTG.

**Fig. 2:**
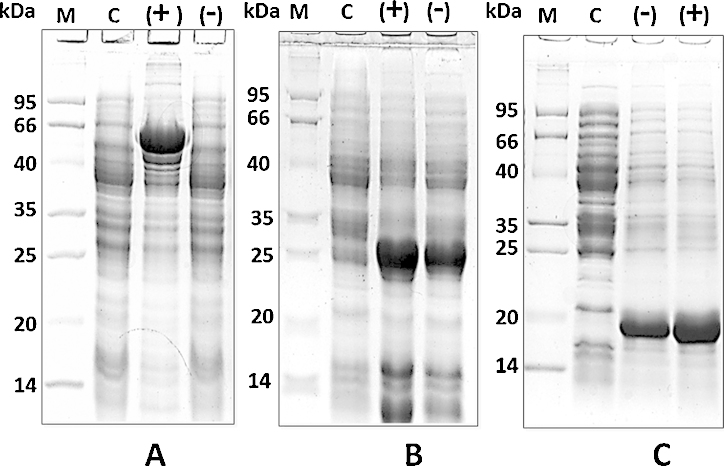
Comparison between normal induced protein and protein induced in presence of 3% ethanol: (A) MEX67 protein: Lane 1, Molecular weight markers (kDa). Lane 2, MEX67 un-induced control. Lane 3, MEX67 induced in presence of 3% ethanol (+). Lane 4, MEX67 induced in absence of 3% ethanol (−). (B) RPB5 protein: Lane 1, Molecular weight markers (kDa). Lane 2, RPB5 un-induced control. Lane 3, RPB5 induced in presence of 3% ethanol (+). Lane 4, RPB5 induced in absence of 3% ethanol (−). (C) RPB8 protein: Lane 1, Molecular weight markers (kDa). Lane 2, RPB8 un-induced control. Lane 3, RPB8 induced in absence of 3% ethanol (−). Lane 4, RPB8 induced in presence of 3% ethanol (+). Protein samples were separated by 12% SDS-PAGE and stained with Coomassie brilliant blue (CBB).

**Fig. 3:**
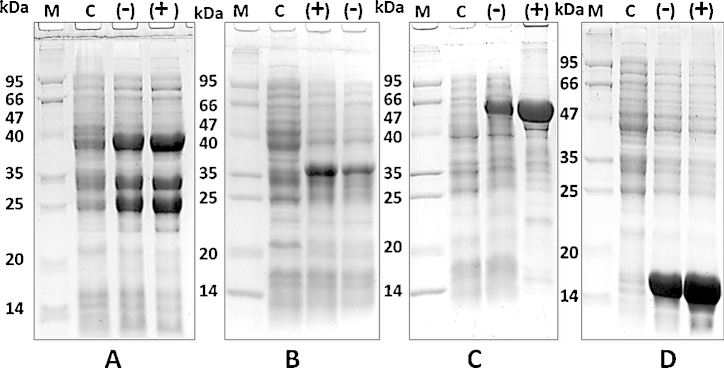
Comparison between normal induced proteins and proteins induced in presence of 3% ethanol: (A) RPB11 protein: Lane 1, Molecular weight markers (kDa). Lane 2, RPB11 un-induced control. Lane 3, RPB11 induced in absence of 3% ethanol (−). Lane 4, RPB11 induced in presence of 3% ethanol (+). (B) Aβ fusion protein: Lane 1, Molecular weight markers (kDa). Lane 2, Aβ un-induced control. Lane 3, Aβ induced in presence of 3% ethanol (+). Lane 4, Aβ induced in absence of 3% ethanol (−) [10]. (C) GlnRS protein: Lane 1, Molecular weight markers (kDa). Lane 2, GlnRS un-induced control. Lane 3, GlnRS induced in absence of 3% ethanol (−). Lane 4, GlnRS induced in presence of 3% ethanol (+). (D) RPB9 protein: Lane 1, Molecular weight markers (kDa). Lane 2, RPB9 un-induced control. Lane 3, RPB9 induced in absence of 3% ethanol (−). Lane 4, RPB9 induced in presence of 3% ethanol (+). Protein samples were separated by 12% SDS-PAGE and stained with CBB.

In our laboratory, we often use the present protocol to enhance the recombinant protein expression in *E. coli*. SDS-PAGE results show the enhancement in recombinant protein expression in the presence of 3% ethanol in comparison to un-induced control and induced recombinant protein expression in the absence of 3% ethanol. We have taken different un-related recombinant proteins from various organisms to support our reported method, which is exceedingly helpful for enhancing the over-expression of recombinant proteins. It works with both T5 (pQE series) and T7 (pET series) type bacterial promoters (Table 1). The method is validated in our laboratory with several recombinant proteins, including MEX67, RPB5, RPB8, RPB11 (from *Saccharomyces cereviseae*), amyloid-beta peptide (Aβ-42 peptide) fusion protein and glutaminyl-tRNA synthetase (GlnRS, from *Fasciola gigantica*). MEX-67 is a 67 kDa poly(A)-RNA binding protein involved in the export of nuclear mRNA and is a component of the nuclear pore and ortholog of human TAP [1], [2], [3], [4]. We observed that the MEX67 protein expression studied under normal IPTG induction demonstrated negligible difference in the level of proteins in comparison to the un-induced control fraction. Optimization was performed with various osmolytes and also with ethanol (Fig. 1). Best expression was found in the induced fraction treated with 3% ethanol (v/v) in the culture media [5] (Fig. 2A). RPB5 (∼25 kDa), one of the small subunit of *S. cerevisiae* is required for yeast cell viability and play a central role in RNA transcription as it is present in all the 3 eukaryotic RNA polymerases [6]. The increase in RPB5 protein expression is shown in Fig. 2B [7]. The 16 kDa RPB8 subunit is essential for cell viability in *S. cerevisiae*, however, its function remains unknown [8]. The difference between RPB8 protein expression in the presence and absence of 3% ethanol is shown in Fig. 2 C. RPB11 fusion protein with GST (∼40 kDa) from *S. cerevisiae* is a RNA polymerase II subunit that is a part of the core element with the central large cleft. RPB11 also seems to be involved transcript termination [9]. Recombinant RPB11 shows increased expression in the presence of ethanol in comparison to normal condition without ethanol as shown in Fig. 3A. Similarly, recombinant Aβ peptide fusion protein (∼30 kDa) showed increased fold of expression in the presence of 3% ethanol in comparison to a condition where ethanol was not present [10] (Fig. 3B). A class I tRNA synthetase, FgGlnRS (∼64 kDa) was recombinantly expressed in high quantity in the presence of ethanol as shown in Fig. 3C. RPB9 from *S. cerevisiae* is involved in the selection of the transcription initiation site, control of fidelity and transcription coupled DNA repair [11]. Like other recombinant proteins, RPB9 (∼14 kDa) also showed increased level of expression in the presence of 3% ethanol (Fig. 3D). Method validation in a wide range of proteins from different organisms provides additional support to the success of our protocol.

## Additional information

There is no report available in the literature where ethanol was used to enhance the expression of recombinant protein by many folds. Increased expression of the recombinant proteins under ethanol treatment is unique and interesting. Ethanol is an amphipathic molecule and can affect the cellular environment of the cell to a large extent by making changes in the membrane fluidity [12], [13], membrane transport [12], membrane lipid composition [12], [14], assembly of membrane proteins [15], [16]. These changes may influence the membrane associated phenomenon, such as DNA replication, leading to the enhancement of DNA synthesis [17]. We propose that enhancement of DNA synthesis results in gene amplification that may enhance the synthesis of inducible proteins in ethanol-treated cells.

Recombinant proteins are necessary for biotechnology and pharmaceutical industries. They are also required for various R & D programme. Structural, functional or biochemical characterization of proteins requires a large amount of purified recombinant proteins. Few methods have been reported which can improve the expression of recombinant protein, but they cannot be universally utilized in comparison to our protocol. We have compared the expression fold of proteins with several osmolytes such as sorbitol, glucose, inositol and sucrose, and compared with that of ethanol. Our results showed that recombinant protein expression under the presence of ethanol gives higher fold of expression compared to osmolytes [5].

Other than enhancing expression of recombinant protein, addition of ethanol to the growth media can mimic the heat-shock response in *E. coli* [18], [19], [20] that can also help in enhancing the solubility of recombinant proteins. Few reports also suggested that the presence of ethanol results in enhancement of protein stability through stabilizing the native state of proteins [21], [22], [23], [24], [25]. Understanding the exact molecular mechanism behind the increase in recombinant protein expression using ethanol can be of enormous applications in future.

## Conflict of interest

The authors declare that there are no conflicts of interest.

## Figures and Tables

**Table 1:** Table showing the different vectors and host strains used for production of the proteins.

Protein name	Vector	Host cells	Antibiotic
MEX67	pQE30	SG13009	Ampicillin, kanamycin
RBP5	pET-28a(+)	BL21(DE3)	Kanamycin
RPB8	pET-28a(+)	BL21(DE3)	Ampicillin
RBP9	pQE30	SG13009	Ampicillin, kanamycin
RPB11	pET-41a(+)	BL21(DE3)	Kanamycin
Amyloid-beta peptide	pET-41a(+)	BL21(DE3)	Kanamycin
Glutaminyl-tRNA synthetase	pET-23a(+)	BL21(DE3)	Ampicillin
